# Urethral Amyloidosis in a Patient with IgA Nephropathy After Renal Transplant

**DOI:** 10.7759/cureus.1296

**Published:** 2017-05-30

**Authors:** Nicholas Ojile, Mona Brake

**Affiliations:** 1 Internal Medicine, University of Kansas School of Medicine - Wichita

**Keywords:** amyloidosis, iga nephropathy, urethra

## Abstract

Urethral amyloidosis is a rare condition that can cause hematuria and urinary obstruction symptoms. While there is no established association between immunoglobulin A (IgA) nephropathy and secondary amyloidosis, which is typically found in chronic inflammatory conditions, it is hypothesized that IgA nephropathy may be a systemic condition with inflammatory mediators. We present a case of urethral amyloidosis in a patient with IgA nephropathy who previously received a renal transplant.

## Introduction

Amyloidosis is an uncommon condition characterized by the deposition of abnormal β- sheet fibrillar proteins in various organs including the kidneys, spleen, and liver [1-2]. It is found less commonly in the genitourinary tract, particularly in the urethra; fewer than 60 cases of urethral amyloidosis have been described in the literature [3-4]. Urethral amyloidosis can present at any age; but, it is mostly found in males with initial presenting symptoms including hematuria, dysuria, urethral discharge, penile induration or masses, and gross urethral bleeding [4-5]. While urethral amyloidosis is a rare condition, there is no known association between immunoglobulin A (IgA) nephropathy and amyloidosis.

## Case presentation

A 35-year-old male presented to the urology clinic with complaints of intermittent gross hematuria (for the previous two days) associated with dysuria and perineal discomfort without penile discharge. He denied urinary hesitancy or intermittency. His past medical history includes IgA nephropathy diagnosed nine years before this presentation. At the time of his IgA nephropathy diagnosis, he presented with gross hematuria and was in end-stage renal disease. He received a living-unrelated donor renal transplant the same year after several months of hemodialysis with no history of graft rejection. He continued to maintain a good graft function, with a serum creatinine of 1.3-1.5 mg/dL. His current medications include sirolimus 1 mg daily, prednisone 5 mg daily, mycophenolic acid 360 mg twice daily, and cholecalciferol 1000 units daily. His family history was significant for IgA nephropathy in his father. Vital signs were within normal limits and the physical examination was remarkable for a palpable transplanted kidney in the left lower quadrant.

Evaluation of the new hematuria included a urine analysis showing red blood cells too numerous to count; the specimen was grossly bloody and there was no proteinuria. Urine culture was negative for bacterial growth. Further evaluation with a computed tomography (CT) scan of the abdomen and pelvis showed atrophic native bilateral kidneys with minimal calcification.

**Figure 1:**
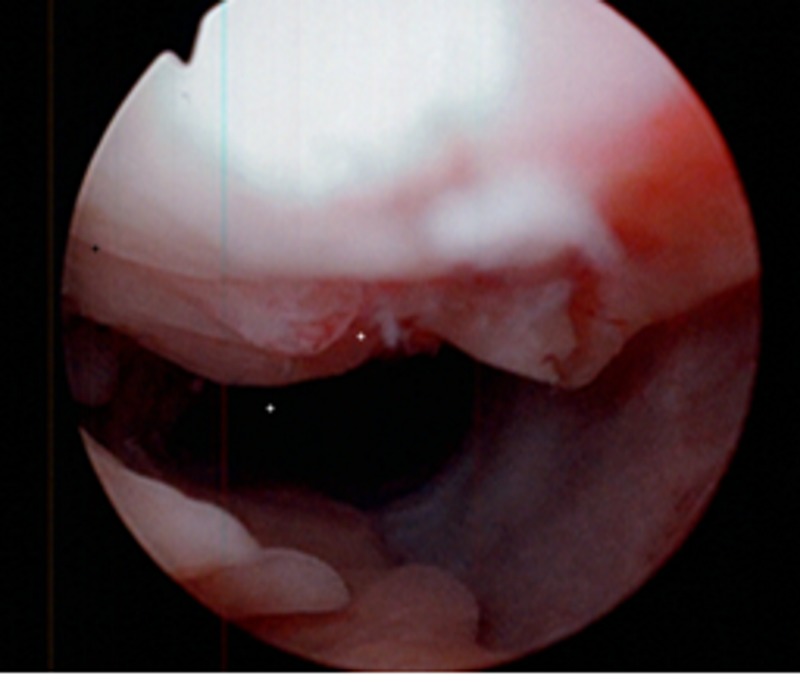
Cystoscopic view of the anterior urethral amyloid tissue

**Figure 2:**
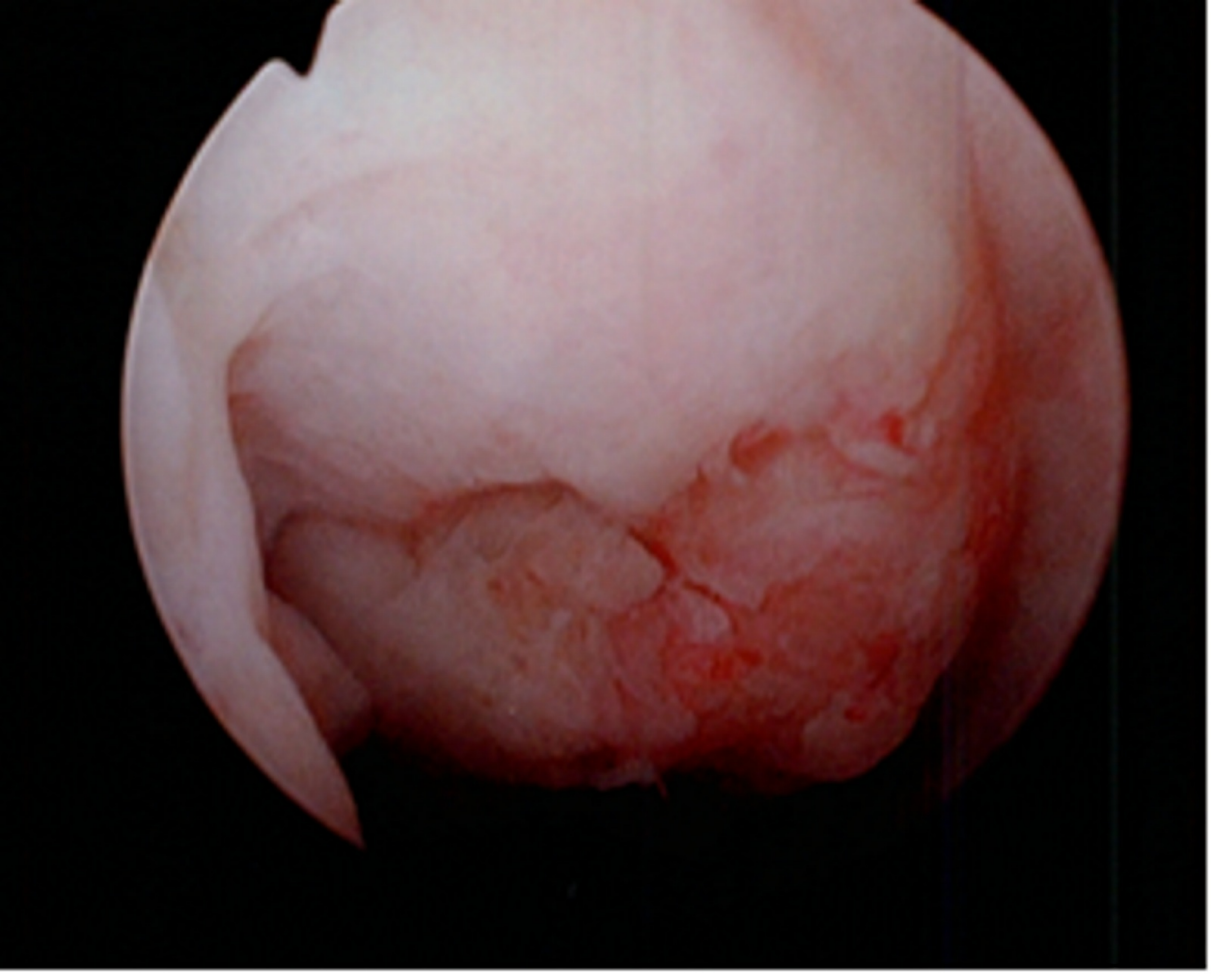
A closer cystoscopic view of the ulcerated amyloid tissue

Without a clear source of the hematuria, he subsequently underwent flexible cystoscopy. An anterior ulcerated mass was noted 3 cm proximal to the urethral meatus (Figures 1-2). The scope was maneuvered around the mass and the remainder of the cystoscopy showed no further abnormalities or another source of hematuria. The mass was resected and measured 1.7 x 1.9 x .5 cm. The amorphous mass was strongly positive for amyloid P protein (AP) but negative for amyloid A (AA), and histopathology showed chronic inflammatory changes. The patient was referred to oncology and further hematologic workup (including normal liver chemistries, serum and urine protein electrophoresis, and kappa/lamda light chain ratio) ruled out primary amyloidosis and confirmed secondary amyloidosis. The patient was recommended for continued observation after the resection.

## Discussion

Urethral amyloidosis is a challenging diagnosis because it can mimic other conditions presenting with gross hematuria including urethral malignancy and IgA nephropathy [3, 6]. The lesion can be found with cystoscopy and in most cases of localized urethral amyloid tissue, the only treatment required is local excision of the tissue. Recurrence of a urethral amyloid is uncommon [4]. Once an amyloid tissue has been identified on histopathology, further workup to distinguish primary (also called systemic amyloidosis) versus secondary amyloidosis is recommended [3].

Secondary amyloidosis is usually associated with chronic inflammatory conditions such as rheumatoid arthritis, inflammatory bowel disease, and chronic infections [1-2]. In a review of urethral amyloidosis case reports, known inflammatory conditions such as gonorrheal urethritis were identified in several cases, but a majority of cases had no associated inflammatory conditions [4]. To our knowledge, there is no known association between IgA nephropathy and amyloid deposition, although IgA nephropathy may have a systemic inflammatory component that could contribute to the development of amyloidosis. A proposed mechanism is on a cellular level; deposition of IgA may lead to the release of pro-inflammatory mediators which leads to oxidative stress resulting in kidney damage leading to end-stage renal disease [6]. Additionally, there may be activation of complement component 3 (C3) leading to further local cellular injury, although this may be independent of systemic complement activation [6-7]. 

It is hypothesized that IgA nephropathy may represent a chronic systemic disease because it is known to recur in patients who have received a renal transplant, as initially suspected in this patient’s case [6]. Additionally, IgA nephropathy is associated closely with Henoch-Schonlein purpura (HSP), which is a systemic disorder that not only has mesangial IgA deposits but also IgA deposition in the skin. It is thought that perhaps the two diseases are a spectrum [7]. The presence of IgA nephropathy in our patient with urethral amyloidosis may be incidental, but his medical record did not show a history of any other chronic inflammatory disorders and he had never been diagnosed with urethritis but had been empirically treated for possible prostatitis on his initial presentation to the urology clinic. As a result, there may be an association between the IgA nephropathy and urethral amyloidosis.

## Conclusions

Urethral amyloidosis is a challenging diagnosis to make and a rare cause of hematuria that can present with obstructive symptoms. Local excision is usually sufficient for treatment and biopsy can distinguish it from urethral malignancy. Subsequent hematologic evaluations can determine primary versus secondary amyloidosis in a patient with urethral amyloid deposits. IgA nephropathy may be a new association with amyloidosis.
